# Author Correction: Learning the natural history of human disease with generative transformers

**DOI:** 10.1038/s41586-025-09879-y

**Published:** 2025-11-12

**Authors:** Artem Shmatko, Alexander Wolfgang Jung, Kumar Gaurav, Søren Brunak, Laust Hvas Mortensen, Ewan Birney, Tom Fitzgerald, Moritz Gerstung

**Affiliations:** 1 Division of AI in Oncology, German Cancer Research Centre DKFZ, Heidelberg, Germany; 2 European Molecular Biology Laboratory, European Bioinformatics Institute EMBL-EBI, Hinxton, UK; 3 Faculty of Biosciences, Heidelberg University, Heidelberg, Germany; 4 Novo Nordisk Foundation Center for Protein Research, Faculty of Health and Medical Sciences, University of Copenhagen, Copenhagen, Denmark; 5 Statistics Denmark, Copenhagen, Denmark; 6 Department of Biosystems Science and Engineering, ETH Zurich, Basel, Switzerland; 7 Department of Public Health, University of Copenhagen, Copenhagen, Denmark; 8 ROCKWOOL Foundation, Copenhagen, Denmark; 9 Faculty of Mathematics and Computer Science, Heidelberg University, Heidelberg, Germany; 10 Robert Bosch Center for Tumor Diseases, Stuttgart, Germany; 11 Medical Faculty, Eberhard-Karls-University, Tübingen, Germany; 12 University Hospital Tübingen, Tübingen, Germany

**Keywords:** Diseases, Risk factors, Computational science

Correction to: *Nature* 10.1038/s41586-025-09529-3 Published online 17 September 2025

In the version of this article initially published, in the Methods “Exponential waiting time model” section, there was a typographical error in the equation now reading “loss_*j*_ = – log *P*(*j*) = cross_entropy(logits, tokens),” where a minus sign originally appeared before the cross-entropy term. The equation has been amended in the HTML and PDF versions of the article.

