# Supplementary material for: Learning the natural history of human disease with generative transformers
Source: Nature. 2025 Sep 17;647(8088):248–56. doi: 10.1038/s41586-025-09529-3 (PMC12589094; doi:10.1038/s41586-025-09529-3)
Supplement: Supplementary file 1 — Supplementary Figs. 1–12, Supplementary Table 1 and Supplementary Discussion. [file 41586_2025_9529_MOESM1_ESM.pdf]

nature portfolio

https://doi.org/10.1038/s41586-025-09529-3

Supplementary information

Learning the natural history of human disease with generative transformers

In the format provided by the authors and unedited

Supplementary information

Supplementary information 1
Supplementary figures 2
Supplementary Figure 1. AUCs for model replicates. 2
Supplementary Figure 2. Ablation study. 3
Supplementary Figure 3. Evaluation of Delphi-2M's performance for future predictions. 4
Supplementary Figure 4. Longitudinal testing on UK Biobank data. 6
Supplementary Figure 5. Sampling health trajectories for long-term predictions. 7
Supplementary Figure 6. Delphi-sampled trajectories are unique and allow for training models without accessing original data. 8
Supplementary Figure 7. Analysis of estimated influences among diagnoses. 10
Supplementary Figure 8. Attention maps indicate temporal effects of prior diagnoses. 11
Supplementary Figure 9. External validation using Danish registries. 12
Supplementary Figure 10. Analysis of AUC variance across different subgroups for ethnicity and deprivation. 14
Supplementary Figure 11. Stratification analysis for generated trajectories. 15
Supplementary Figure 12. ICD10 disease token biases. 16
Supplementary tables 17
Supplementary Table 1. Ablation results for Delphi-2M. 17
Supplementary Table 2. Dataset information and AUC values for individual diseases. 17
Supplementary Table 3. Method comparison results. 17
Supplementary Table 4. Subgroup-related biases in Delphi. 17
Supplementary Table 5. Used UK Biobank data fields. 17
Supplementary discussion 18
Ethnicity and deprivation-based biases in Delphi 18

Supplementary figures

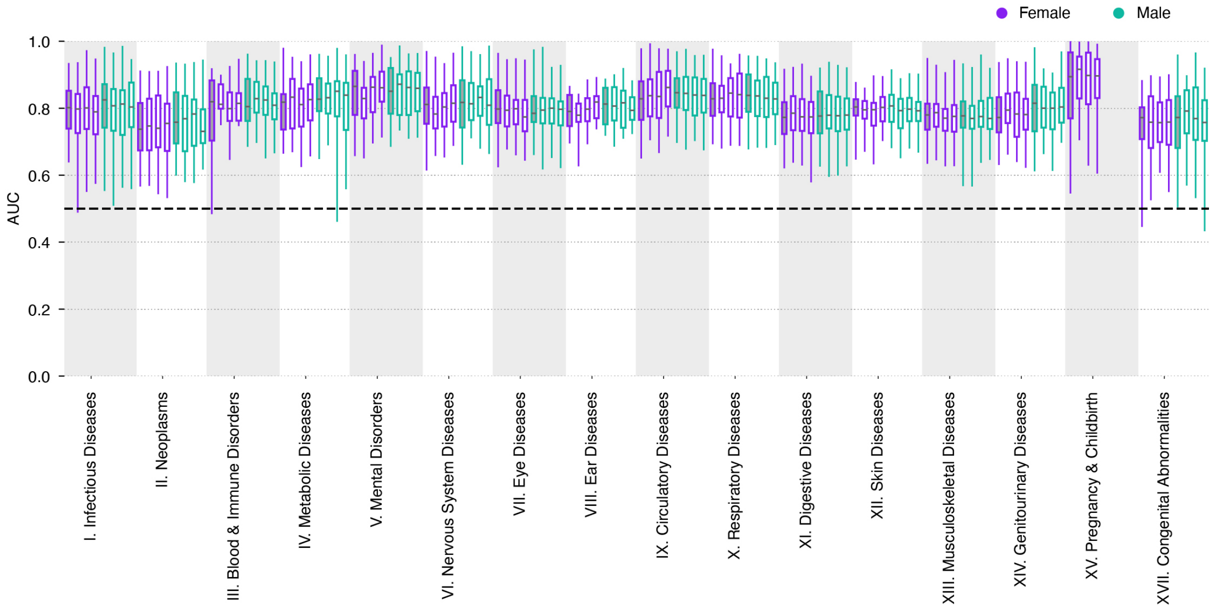

Supplementary Figure 1. AUCs for model replicates.

Age-stratified per-disease AUC (y-axis) distributions grouped by disease chapter (y-axis). Age-stratified AUCs are calculated by averaging AUCs from 5-year age groups ranging from 40 to 80 years of age. Shown are data for n=733 diagnoses for males and n=803 diagnoses for females with at least 1 age bin with 2 occurrences. Colours denote the self-reported sex. The first (shaded) box plot in each group shows values for the original Delphi-2M model, followed by 3 technical replicates, each trained on different non-intersecting train-validation data split. The boxplots feature the median as the center line, the box from the first to the third quartile and the whiskers for 0.025 and 0.975 quantiles.

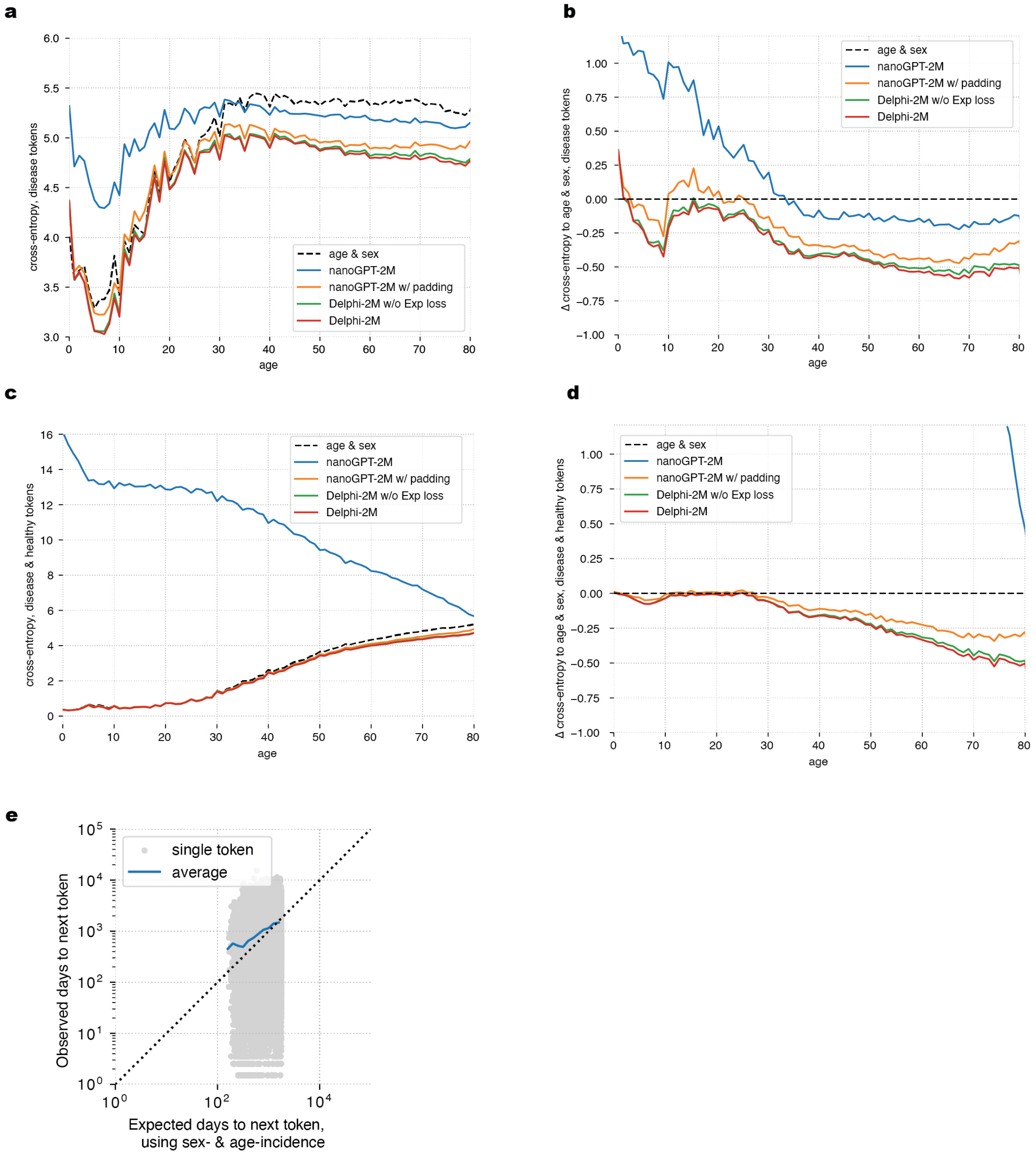

Supplementary Figure 2. Ablation study.

a, Average validation cross-entropy loss for disease tokens (y-axis) as a function of age (x-axis). **b**, Cross-entropy loss for disease tokens relative to sex- and age-incidence baseline (y-axis) as a function of age (x-axis). **c,** Average cross-entropy loss for disease and healthy tokens (y-axis) in relation to age (x-axis). **d**, Average cross-entropy loss for disease and healthy tokens relative to sex- and age-incidence baseline (y-axis) as a function of age (x-axis). **e**, Accuracy of predicted time-to-event. Shown are observed (y-axis) and expected (x-axis) time to events for each next token prediction (grey dots). The blue line shows the average across consecutive bins of the x-axis.

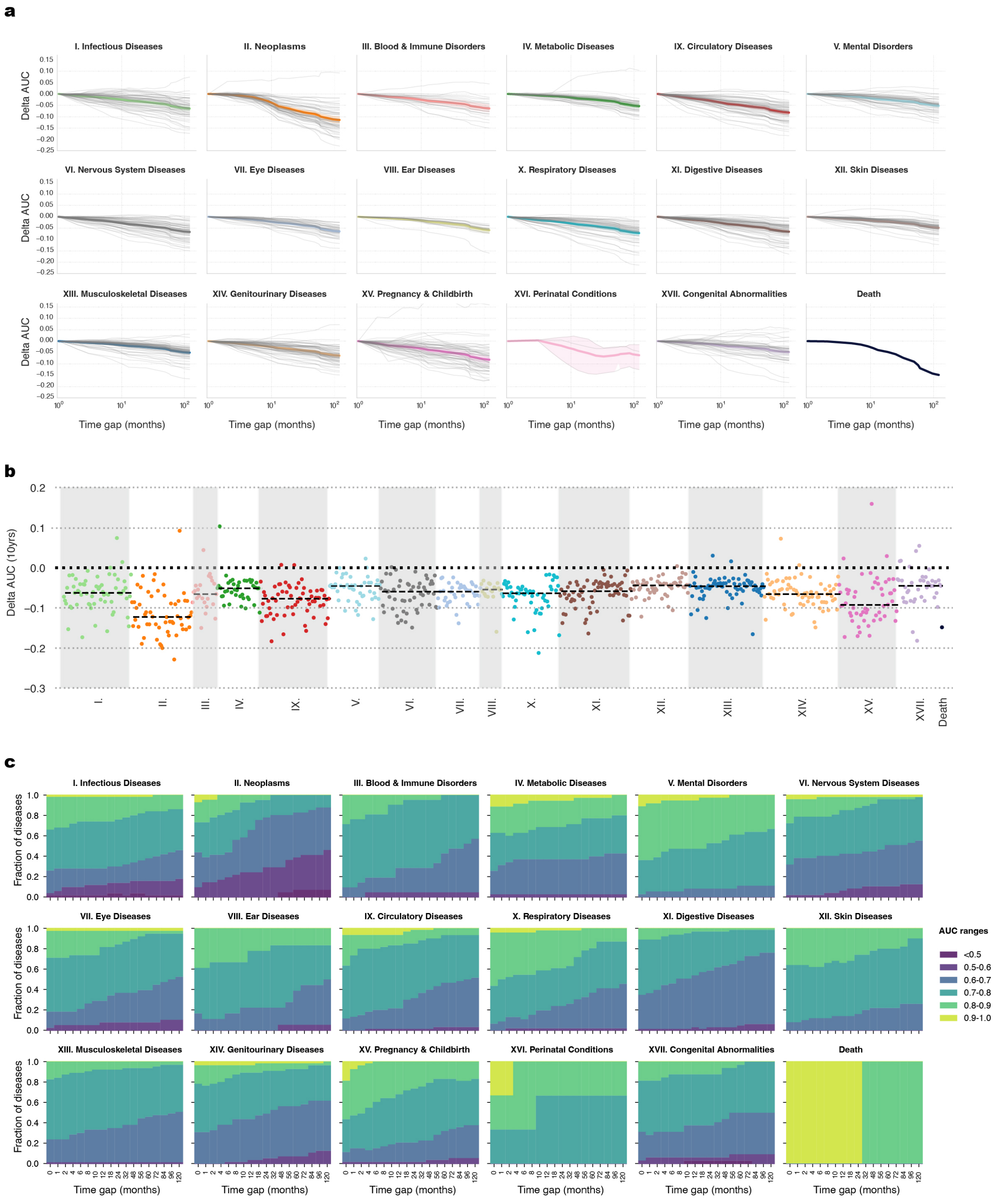

Supplementary Figure 3. Evaluation of Delphi-2M's performance for future predictions. Average validation AUC across 5-year age groups ranging from 40 to 80 years of age, aggregated by the corresponding ICD chapters. **a.** Each grey line represents the AUC for a particular disease when evaluated with different time gaps, with the bold line indicating the per-chapter average. The shaded region indicates the 95% confidence interval for the average. **b.** Same data as in **a**, showing the delta AUC for all diseases with a 10-year time gap compared to the next token AUC. **c,** Stacked bar chart, indicating the fraction of diseases that are predicted with a certain AUC, given a time gap into the future.

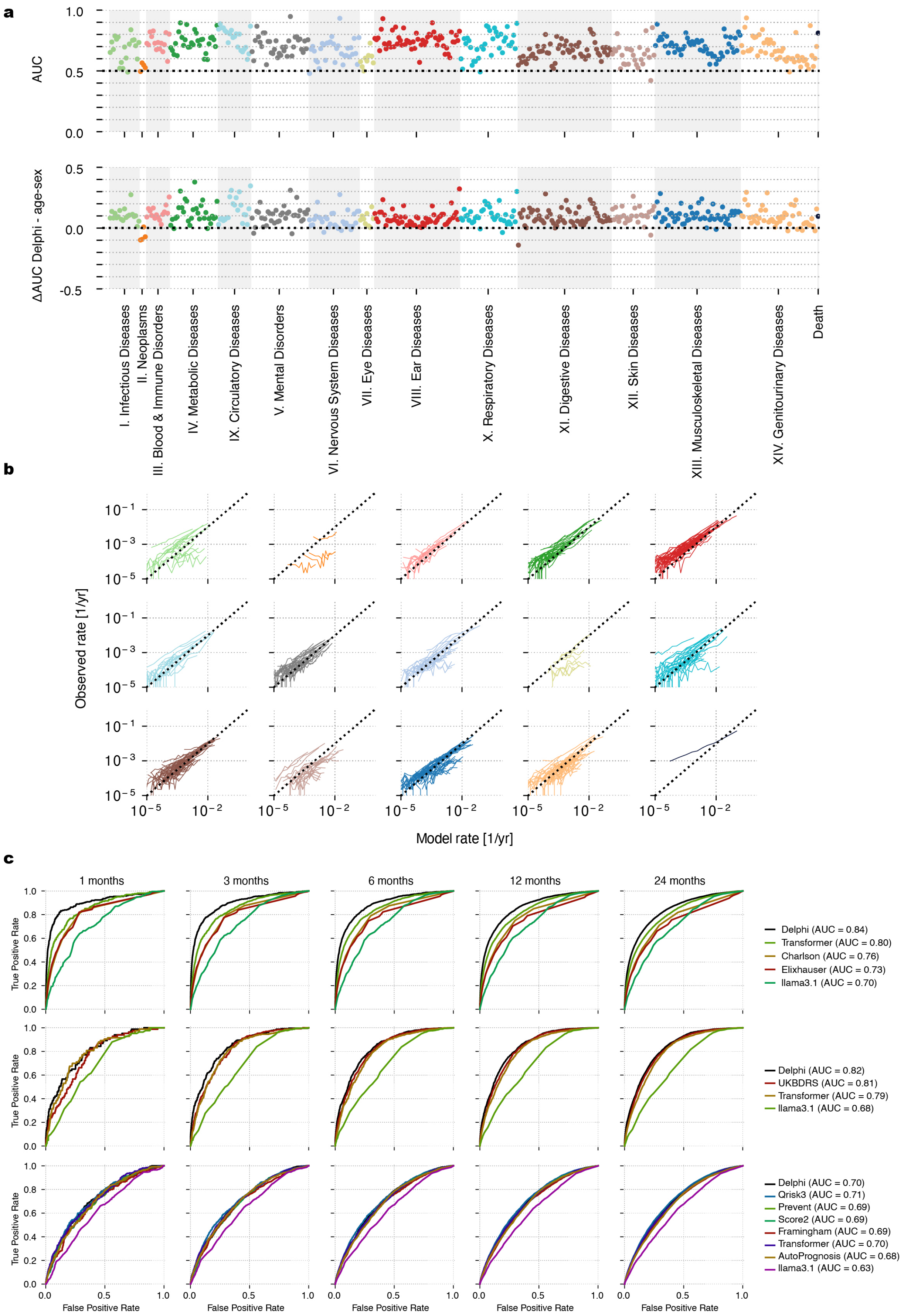
a
AUC
1.0
0.5
0.0
ΔAUC Delphi - age-sex
0.5
0.0
-0.5
I. Infectious Diseases
II. Neoplasms
III. Blood & Immune Disorders
IV. Metabolic Diseases
IX. Circulatory Diseases
V. Mental Disorders
VI. Nervous System Diseases
VII. Eye Diseases
VIII. Ear Diseases
X. Respiratory Diseases
XI. Digestive Diseases
XII. Skin Diseases
XIII. Musculoskeletal Diseases
XIV. Genitourinary Diseases
Death
b
Observed rate [1/yr]
Model rate [1/yr]
c
1 months
3 months
6 months
12 months
24 months
Death
Demantia
CVD
True Positive Rate
False Positive Rate
Delphi (AUC = 0.84)
Transformer (AUC = 0.80)
Charlson (AUC = 0.76)
Elixhauser (AUC = 0.73)
llama3.1 (AUC = 0.70)
Delphi (AUC = 0.82)
UKBDRS (AUC = 0.81)
Transformer (AUC = 0.79)
llama3.1 (AUC = 0.68)
Delphi (AUC = 0.70)
Qrisk3 (AUC = 0.71)
Prevent (AUC = 0.69)
Score2 (AUC = 0.69)
Framingham (AUC = 0.69)
Transformer (AUC = 0.70)
AutoPrognosis (AUC = 0.68)
llama3.1 (AUC = 0.63)

Supplementary Figure 4. Longitudinal testing on UK Biobank data.

a, AUC results of Delphi for each token with at least 25 occurrences coloured by the respective ICD-10 chapter. Predictions are based on UKB adults (50-80 years of age at cutoff) with data up until 30. June. 2020 and evaluated on incidence between 1. July. 2021 - 1. July. 2022. Below are the respective differences in AUC values between Delphi and a UK Biobank age-sex baseline. **b,** Calibration plots split by each ICD-10 chapter. Each line represents an ICD-10 disease evaluated for each decile of the Delphi rate and compared against the observed rate in the population. **c,** Comparison of Delphi against other clinical or machine learning methods for cardiovascular disease, dementia and death (rows) with a varying prediction horizon (columns).

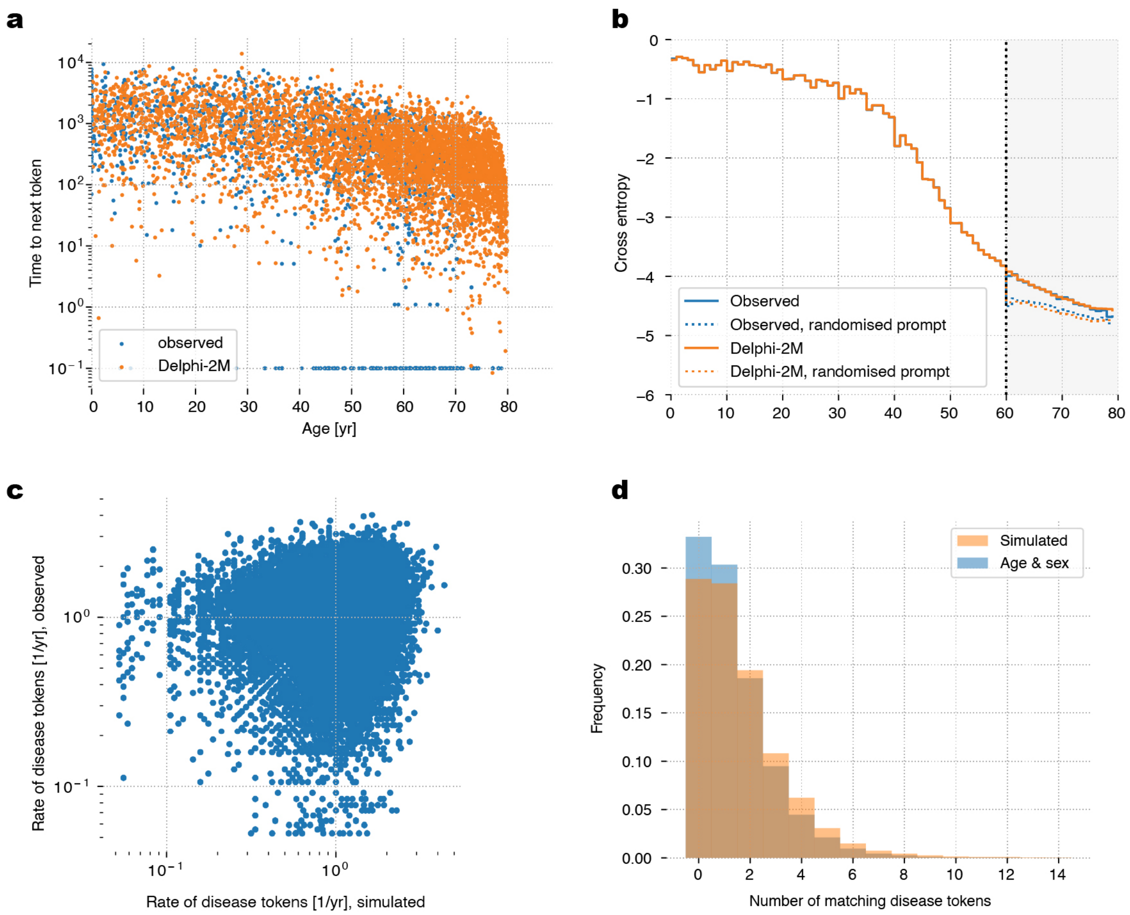

Supplementary Figure 5. Sampling health trajectories for long-term predictions.
a, Simulated (orange) and observed (blue) Delphi times (y-axis) to the next event (y-axis) as a function of age (x-axis). Simulations are from birth. **b,** Shuffling the input sequences (prompts) between individuals of the same sex causes a drop in cross-entropy (y-axis). **c,** Delphi-2M predicted rate of diagnoses (number of simulated tokens/15-year follow-up from 60-75; x-axis) versus the observed rate (y-axis) for each participant (dots) in the same period. Simulations are based on observed trajectories until age 60. **d,** Distribution of the number of predicted tokens using simulations after age 60 (orange) compared to age and sex (blue).

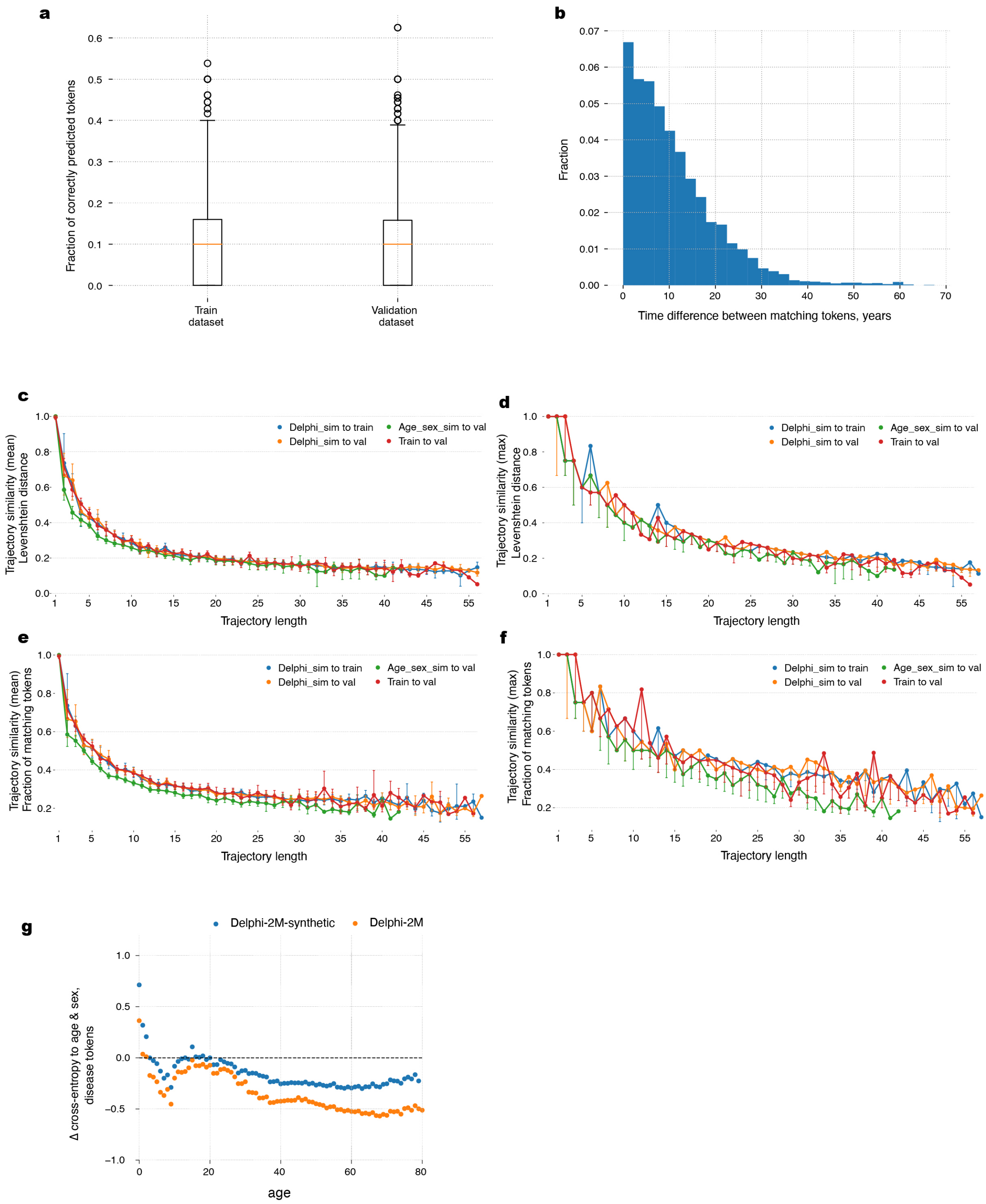

Supplementary Figure 6. Delphi-sampled trajectories are unique and allow for training models without accessing original data.

a, When completing the partial trajectories from the train and validation subsets of the UKB dataset, the fractions (from n=8192 health trajectories) of correctly guessed tokens are the same, indicating that there is no train set overfit. The boxplots feature median as the center line, the box from the first to the third quartile, the whiskers for 1.5x IQR and the outliers. **b,** When sampling from scratch, even the trajectories similar to real ones token-wise have major shifts time-wise. **c-f,** In terms of the fraction of matching tokens and normalised Levenshtein distance, Delphi-sampled trajectories are closer to real ones compared to age-sex sampled. Error bars indicate 95% CI, closest trajectories in the entire dataset were calculated for random n=2048 trajectories. **g,** Delphi-2M-synthetic, a model trained on trajectories sampled from Delphi-2M, still performs better than the age-sex epidemiological baseline.

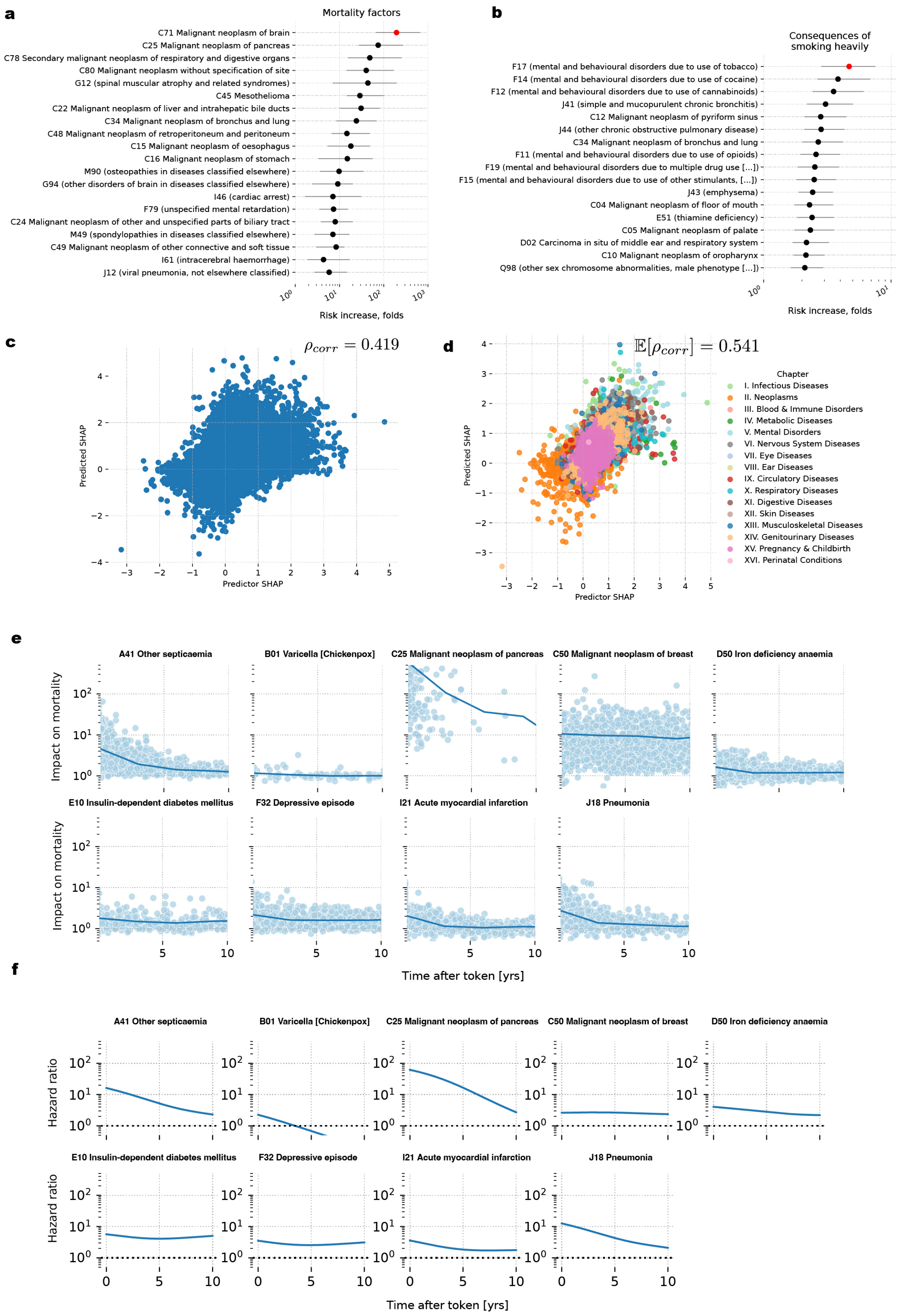
a
Mortality factors
C71 Malignant neoplasm of brain
C25 Malignant neoplasm of pancreas
C78 Secondary malignant neoplasm of respiratory and digestive organs
C80 Malignant neoplasm without specification of site
G12 (spinal muscular atrophy and related syndromes)
C45 Mesothelioma
C22 Malignant neoplasm of liver and intrahepatic bile ducts
C34 Malignant neoplasm of bronchus and lung
C48 Malignant neoplasm of retroperitoneum and peritoneum
C15 Malignant neoplasm of oesophagus
C16 Malignant neoplasm of stomach
M90 (osteopathies in diseases classified elsewhere)
G94 (other disorders of brain in diseases classified elsewhere)
I46 (cardiac arrest)
F79 (unspecified mental retardation)
C24 Malignant neoplasm of other and unspecified parts of biliary tract
M49 (spondylopathies in diseases classified elsewhere)
C49 Malignant neoplasm of other connective and soft tissue
I61 (intracerebral haemorrhage)
J12 (viral pneumonia, not elsewhere classified)
Risk increase, folds
b
Consequences of smoking heavily
F17 (mental and behavioural disorders due to use of tobacco)
F14 (mental and behavioural disorders due to use of cocaine)
F12 (mental and behavioural disorders due to use of cannabinoids)
J41 (simple and mucopurulent chronic bronchitis)
C12 Malignant neoplasm of pyriform sinus
J44 (other chronic obstructive pulmonary disease)
C34 Malignant neoplasm of bronchus and lung
F11 (mental and behavioural disorders due to use of opioids)
F19 (mental and behavioural disorders due to multiple drug use [...])
F15 (mental and behavioural disorders due to use of other stimulants, [...])
J43 (emphysema)
C04 Malignant neoplasm of floor of mouth
E51 (thiamine deficiency)
C05 Malignant neoplasm of palate
D02 Carcinoma in situ of middle ear and respiratory system
C10 Malignant neoplasm of oropharynx
Q98 (other sex chromosome abnormalities, male phenotype [...])
Risk increase, folds
c
$\rho_{corr} = 0.419$
Predicted SHAP
Predictor SHAP
d
$\mathbb{E}[\rho_{corr}] = 0.541$
Chapter
I. Infectious Diseases
II. Neoplasms
III. Blood & Immune Disorders
IV. Metabolic Diseases
V. Mental Disorders
VI. Nervous System Diseases
VII. Eye Diseases
VIII. Ear Diseases
IX. Circulatory Diseases
X. Respiratory Diseases
XI. Digestive Diseases
XII. Skin Diseases
XIII. Musculoskeletal Diseases
XIV. Genitourinary Diseases
XV. Pregnancy & Childbirth
XVI. Perinatal Conditions
Predicted SHAP
Predictor SHAP
e
A41 Other septicaemia
B01 Varicella [Chickenpox]
C25 Malignant neoplasm of pancreas
C50 Malignant neoplasm of breast
D50 Iron deficiency anaemia
E10 Insulin-dependent diabetes mellitus
F32 Depressive episode
I21 Acute myocardial infarction
J18 Pneumonia
Impact on mortality
Time after token [yrs]
f
A41 Other septicaemia
B01 Varicella [Chickenpox]
C25 Malignant neoplasm of pancreas
C50 Malignant neoplasm of breast
D50 Iron deficiency anaemia
E10 Insulin-dependent diabetes mellitus
F32 Depressive episode
I21 Acute myocardial infarction
J18 Pneumonia
Hazard ratio
Time after token [yrs]

Supplementary Figure 7. Analysis of estimated influences among diagnoses.
a, Strongest SHAP predictors of increased mortality risk, based of the SHAP analysis of n=2,531,438 tokens from the validation dataset. Points show the median effect, lines extend to the 25 and 75 percentiles. **b,** Tokens with the largest risk increase, attributed to the "Smoking - high" lifestyle token, based on n=7417 health trajectories with recorded smoking. Points show the median effect, lines extend to the 25 and 75 percentiles. **c,** Scatter plot of pairs "SHAP value (logarithmic fold change) of token X predicting diagnosis Y" and vice versa for all possible pairs of tokens. The value on top denotes the Pearson correlation. **d,** Same as **c**, but restricted to pairs within the same ICD-10 chapters (indicated by colour). **e,** SHAP values (fold change) for mortality (y-axis) as a function of time after diagnosis of 9 different diseases (x-axis). **f,** Nelson-Aalen analysis of the hazard rates for the same diseases shown in **e** confirming trend and magnitude of SHAP effects.

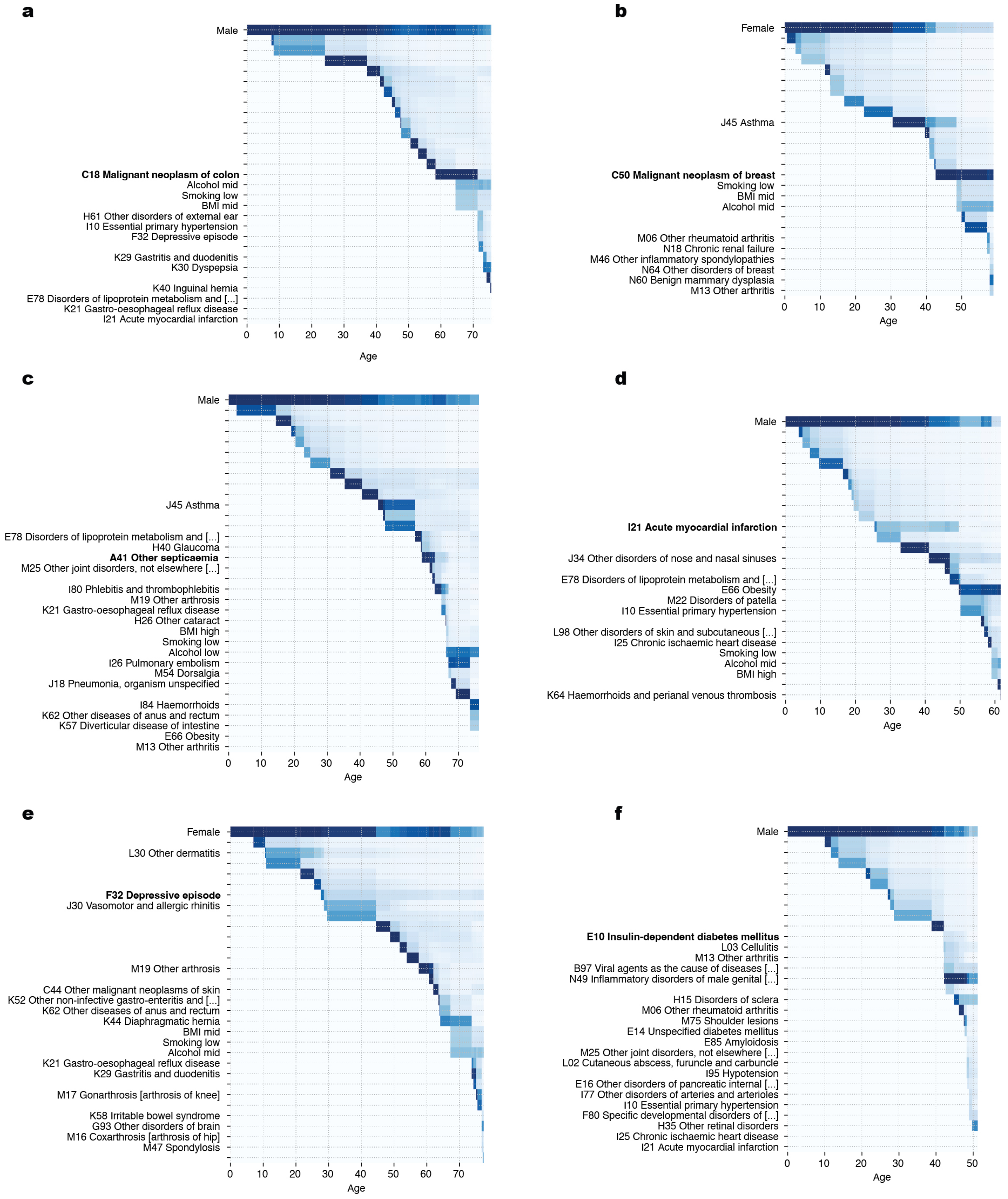

Supplementary Figure 8. Attention maps indicate temporal effects of prior diagnoses.

a-f. Attention weights for 6 selected trajectories representative of the token highlighted on the y-axis. Shown is the maximum attention across the 12 layers and 12 heads utilised by Delphi-2M. Darker values indicate higher attention (range 0-1). Typically, the last (token, age) pair is most strongly attended to, after which attention drops. Sex and selected other inputs are attended to for more extended periods. Rows without labels are “No event” tokens.

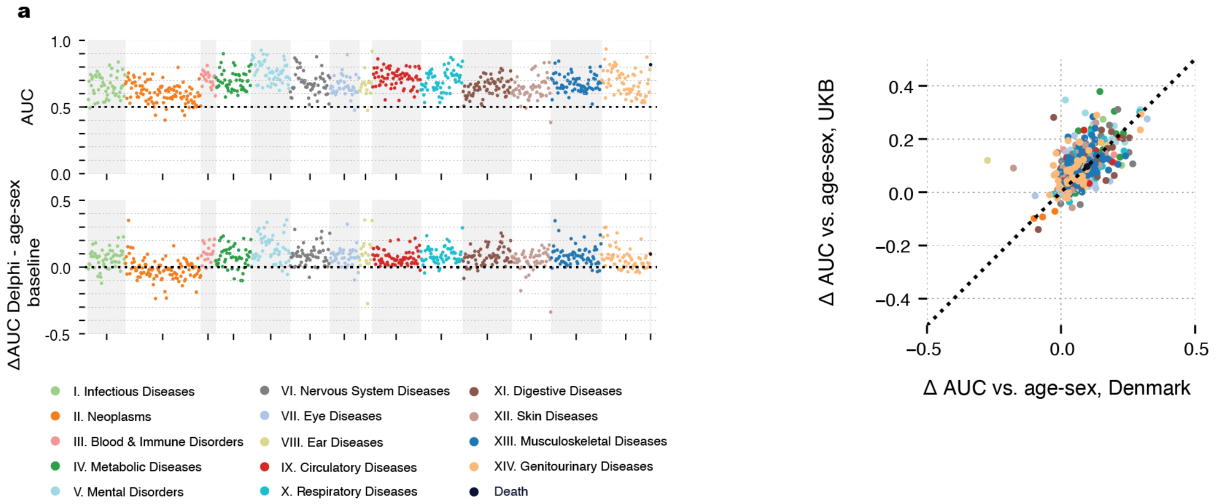

Supplementary Figure 9. External validation using Danish registries.
a, AUC results of Delphi for each token with at least 25 occurrences, coloured by the respective ICD-10 chapter. Predictions are based on Danish adults (50-80 years of age) with data up until 1. Jan. 2016 and evaluated on incidence between 1. Jan. 2017 - 1. Jan. 2018. Below are the respective differences in AUC values between Delphi and an age-sex baseline based on Danish data from 2010-2016. **b,** Comparison between ΔAUC values in the UKB longitudinal testing and the Danish testing with their respective age-sex baseline.

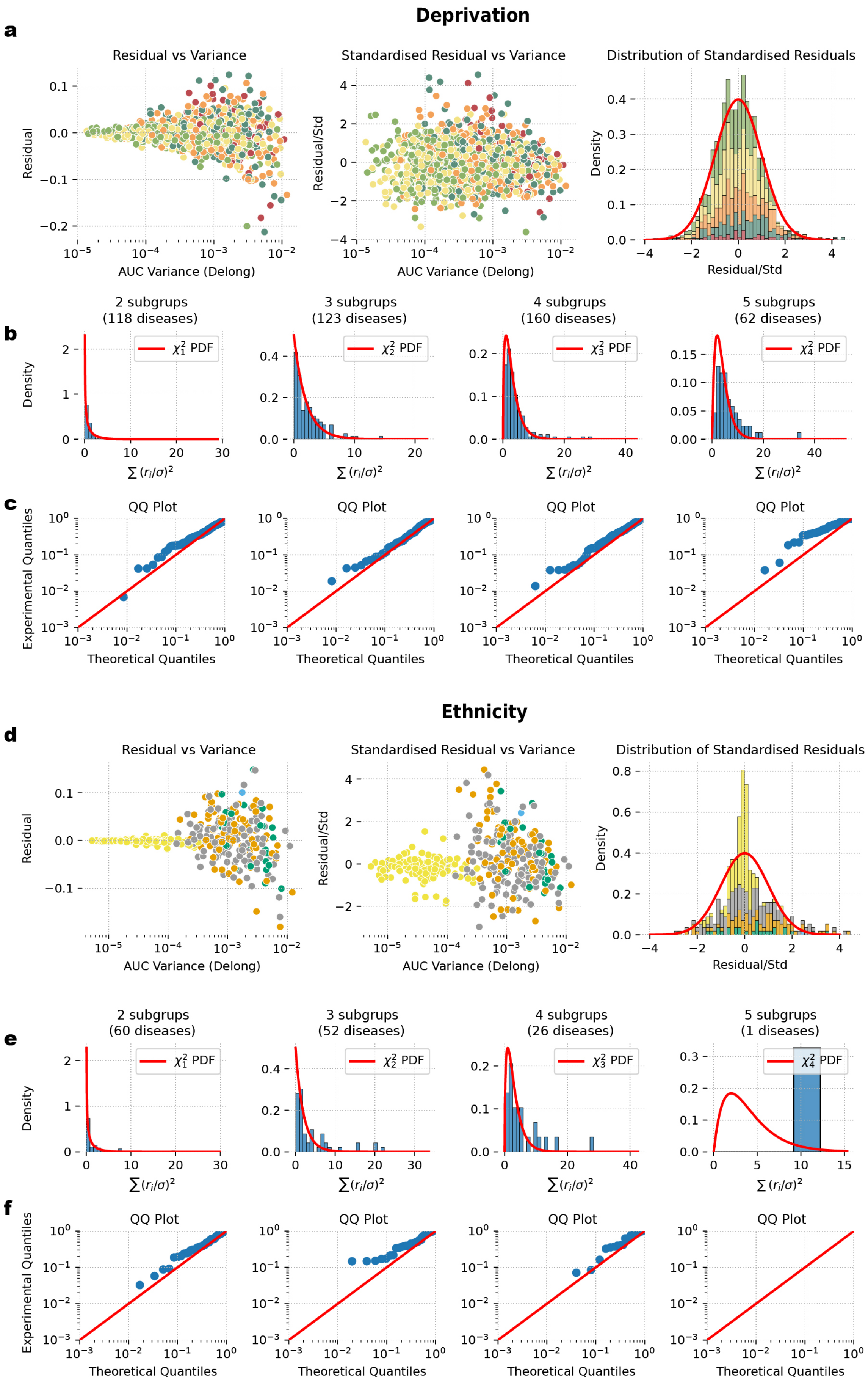
Deprivation
a
Residual vs Variance
Residual
AUC Variance (Delong)
Standardised Residual vs Variance
Residual/Std
AUC Variance (Delong)
Distribution of Standardised Residuals
Density
Residual/Std
b
2 subgrups (118 diseases)
3 subgrups (123 diseases)
4 subgrups (160 diseases)
5 subgrups (62 diseases)
Density
χ^2_1 PDF
χ^2_2 PDF
χ^2_3 PDF
χ^2_4 PDF
$\sum (r_i/\sigma)^2$
c
QQ Plot
Experimental Quantiles
Theoretical Quantiles
Ethnicity
d
Residual vs Variance
Residual
AUC Variance (Delong)
Standardised Residual vs Variance
Residual/Std
AUC Variance (Delong)
Distribution of Standardised Residuals
Density
Residual/Std
e
2 subgrups (60 diseases)
3 subgrups (52 diseases)
4 subgrups (26 diseases)
5 subgrups (1 diseases)
Density
χ^2_1 PDF
χ^2_2 PDF
χ^2_3 PDF
χ^2_4 PDF
$\sum (r_i/\sigma)^2$
f
QQ Plot
Experimental Quantiles
Theoretical Quantiles

Supplementary Figure 10. Analysis of AUC variance across different subgroups for ethnicity and deprivation.

This analysis is described in Methods in more detail. **a, d,** Analysis of AUC residuals for individual subgroups (subgroup_auc - weighted_average_auc). Left: AUC residuals for different diseases and different subgroups vs DeLong-estimated variance. Centre, same as left, but residuals are standardised by dividing by DeLong-estimated standard deviation. Right, distribution of standardised residuals. The red line indicates a standard normal distribution. **b, e,** Distribution of sums of squares of standardised residuals for different diseases. The red line indicates the corresponding Chi-squared distribution. For some diseases, there are not enough cases for certain subgroups, therefore, we treat diseases with different numbers of available subgroups differently. **c, f,** Q-Q plot for squares of standardised residuals for different diseases and the corresponding Chi-squared distribution.

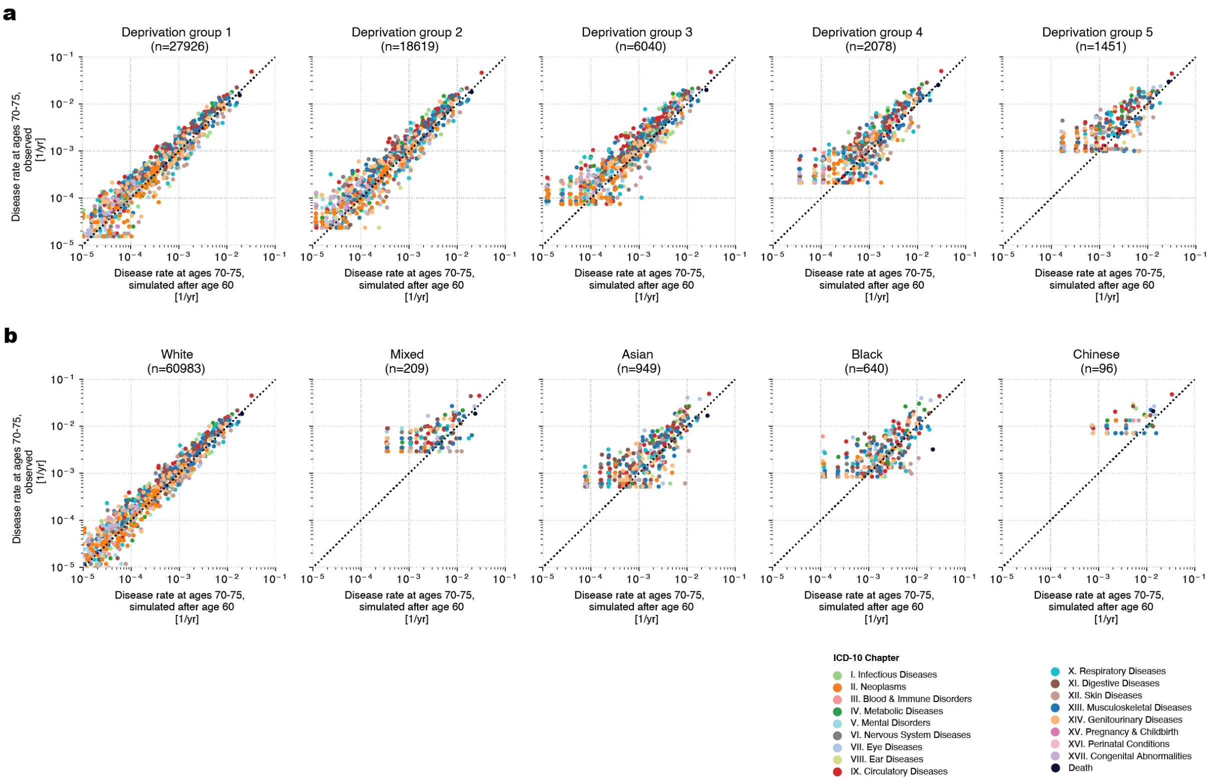

Supplementary Figure 11. Stratification analysis for generated trajectories. Simulated and observed disease rates recorded between ages 70-75 in UK Biobank validation data. Simulations use data until the age of 60. **a,** Scatter plots of simulated and observed rates in participant groups split by Townsend deprivation index. **b,** Scatter plots of simulated and observed disease rates stratified by self-reported ethnicity. Each dot is an ICD-10 diagnosis coloured by the disease chapter.

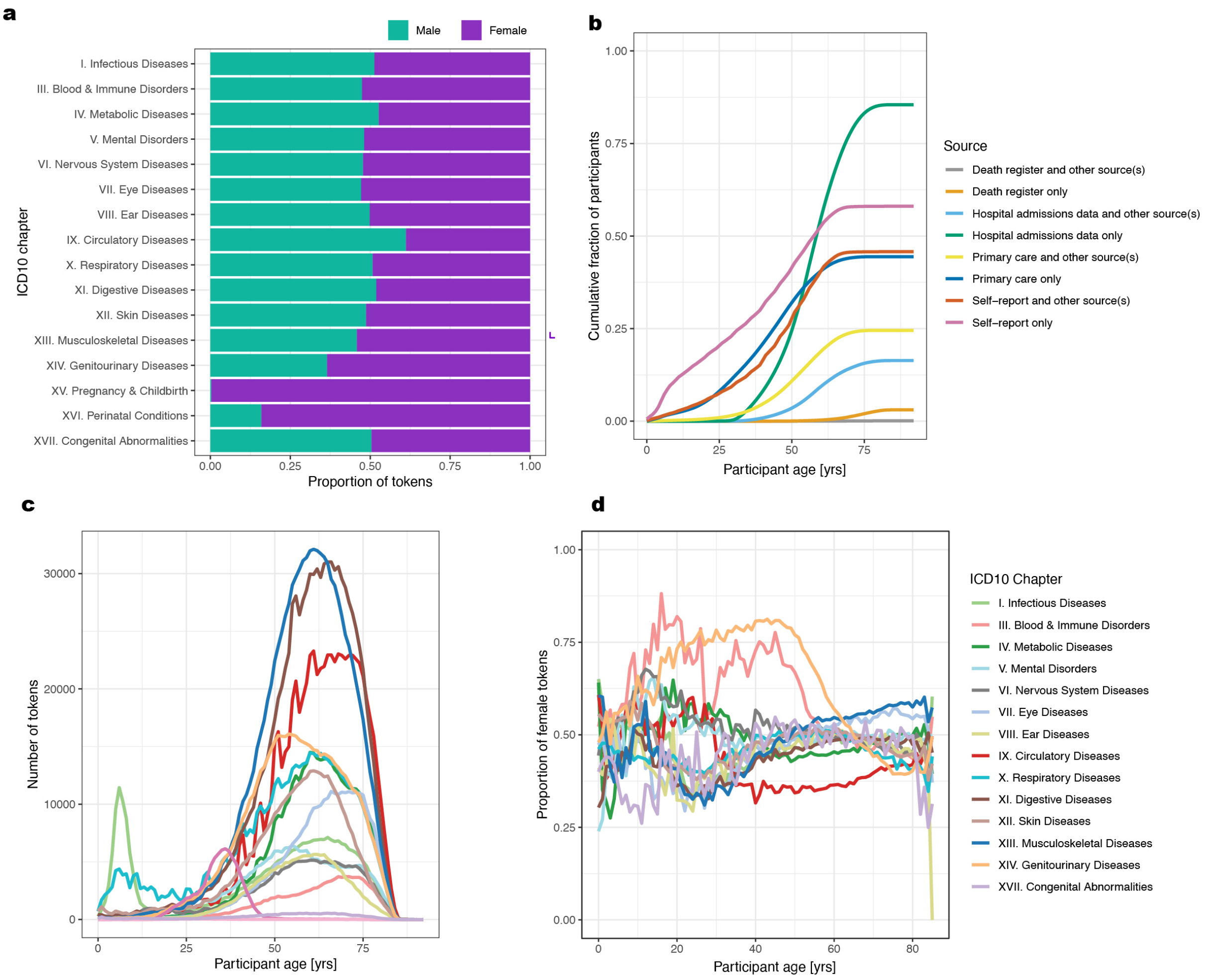

Supplementary Figure 12. ICD10 disease token biases.

a, The proportion of the total disease token set scaled by the number of samples, comparing female and male samples across the ICD-10 chapters. **b,** Participant age against the cumulative sum of the fraction of participants with disease tokens across 8 primary data sources. **c,** Participant age against the total number of disease tokens across ICD-10 chapters. **d,** Participant age against the proportion of tokens in females across the ICD-10 chapters.

Supplementary tables

Loss	Age and Sex	GPT	+ healthy tokens	+ age enc. & masks	+ time loss
Next token class (cross-entropy)	0 (baseline)	–0.206	–0.478	–0.567	**–0.574**
Time to next token (exponential)	0 (baseline)	N/A	N/A	N/A	**–0.19**

Supplementary Table 1. Ablation results for Delphi-2M.
Top. Change in cross-entropy of disease token predictions relative to a baseline derived from age (in years) and sex incidence. **Bottom.** Change in the exponential loss for the waiting time until the next token.

Supplementary Table 2. Dataset information and AUC values for individual diseases.
This table contains information occurrence of diseases in the UK Biobank dataset, their name, ICD code and chapter, token_id used in Delphi-2M and the Delphi-2M AUC for male and female. The table is located in a separate file.

Supplementary Table 3. Method comparison results.
This table contains classification metrics (ROC-AUC, Average Precision Score and Brier Score), evaluated for the task of predicting death, cardiovascular diseases, and dementia for Delphi and other methods, including clinical risk scores and machine learning models. The table is located in a separate file.

Supplementary Table 4. Subgroup-related biases in Delphi.
This table contains list of diseases for which Delphi may be biased towards certain population subgroups, with their AUC values for all subgroups. (**Methods**). The table is located in a separate file.

Supplementary Table 5. Used UK Biobank data fields.
This table contains UKB field IDs for all fields used in the study, including general participant information (sex, lifestyle), first disease occurrences and additional information, used for comparisons to other methods. The table is located in a separate file.

Supplementary discussion

Ethnicity and deprivation-based biases in Delphi

An important consideration of healthcare models is whether predictions are made with similar accuracy in different population groups. The predicted rates of observed diseases are generally comparable between the sexes (**Extended Data Figure 9 a,b**). Across self-reported ethnic backgrounds, however, lower rates for individuals self-reporting “white” are predicted, reflecting the observed disease rates (**Extended Data Figure 9 a,c**). Similarly, Delphi-2M’s predicted rate of disease is elevated for groups with a higher Townsend deprivation index, which mirrors the average number of tokens per individual in this group. (**Extended Data Figure 9 b,d**). For most diseases, the differences in prediction AUCs for different ethnicities and deprivation groups are not statistically significant and stem from variance in AUC estimation, however, with some exceptions (**Extended Data Figure 9e-f, Supplementary Figure 10, Supplementary Table 4**). Simulated trajectories are also found to reproduce the observed disease burden in these groups (**Supplementary Figure 11**). Together, these observations indicate that Delphi-2M’s predictions reflect the differential burden of disease seen across different population subgroups but exhibit mostly comparable discriminatory performance within each stratum. However, due to large confidence intervals for AUC for underrepresented subgroups, studying such biases remains challenging, especially for rare diseases (**Supplementary Figure 9 g-i, Supplementary Figure 10**).
