# Supplementary material for: Learning the natural history of human disease with generative transformers
Source: Nature. 2025 Sep 17;647(8088):248–56. doi: 10.1038/s41586-025-09529-3 (PMC12589094; doi:10.1038/s41586-025-09529-3)
Supplement: Supplementary file 2 — Reporting Summary [file 41586_2025_9529_MOESM2_ESM.pdf]

Corresponding author(s): Ewan Birney, Tom Fitzgerald, Moritz Gerstung

Last updated by author(s): Jul 29, 2025

Reporting Summary

Nature Portfolio wishes to improve the reproducibility of the work that we publish. This form provides structure for consistency and transparency in reporting. For further information on Nature Portfolio policies, see our Editorial Policies and the Editorial Policy Checklist.

Statistics

For all statistical analyses, confirm that the following items are present in the figure legend, table legend, main text, or Methods section.

n/a	Confirmed	
☐	☒	The exact sample size (*n*) for each experimental group/condition, given as a discrete number and unit of measurement
☒	☐	A statement on whether measurements were taken from distinct samples or whether the same sample was measured repeatedly
☐	☒	The statistical test(s) used AND whether they are one- or two-sided<br>*Only common tests should be described solely by name; describe more complex techniques in the Methods section.*
☐	☒	A description of all covariates tested
☐	☒	A description of any assumptions or corrections, such as tests of normality and adjustment for multiple comparisons
☐	☒	A full description of the statistical parameters including central tendency (e.g. means) or other basic estimates (e.g. regression coefficient) AND variation (e.g. standard deviation) or associated estimates of uncertainty (e.g. confidence intervals)
☐	☒	For null hypothesis testing, the test statistic (e.g. *F*, *t*, *r*) with confidence intervals, effect sizes, degrees of freedom and *P* value noted<br>*Give P values as exact values whenever suitable.*
☒	☐	For Bayesian analysis, information on the choice of priors and Markov chain Monte Carlo settings
☒	☐	For hierarchical and complex designs, identification of the appropriate level for tests and full reporting of outcomes
☐	☒	Estimates of effect sizes (e.g. Cohen's *d*, Pearson's *r*), indicating how they were calculated

Our web collection on statistics for biologists contains articles on many of the points above.

Software and code

Policy information about availability of computer code

Data collection: No novel data was collected for this study. The information about datasets used is in the "Data" section.

Data analysis: Python (3.11.9) with packages:
- pytorch (2.3.0)
- numpy (1.26.4)
- matplotlib (3.8.4)
- seaborn (0.13.2)
- shap (0.45.1)
- pandas (2.2.2)
- umap-learn (0.5.6)
- scikit-learn (1.4.2)
- scikit-survival (0.22.2)
- statsmodels (0.14.2)
- autoprognosis (0.1.21)

R (4.2.3) with packages (for plotting):
- reshape2 (1.4.4)
- ggplot2 (3.5.1)
- ggVennDiagram (1.5.2)
- ggpubr (0.6.0)
- RColorBrewer (1.1-3)

- dplyr (1.1.4)
- probcox (0.0.5)

R (4.4.0) with packages (for method comparison):
- CVrisk (1.1.1)
- glmnet (4.1-8)
- mice (3.16.0)
- preventr (0.10.0)
- RiskScorescvd (0.2.0)
- comorbidity (1.1.0)

UKBDRS - https://github.com/MelisAnaturk/dementia_risk_score/tree/main
QRISK3(2017) - https://www.qrisk.org/src.php (version QRISK3-2017)

Custom Delphi code: https://github.com/gerstung-lab/delphi (commit 6132df6)

For manuscripts utilizing custom algorithms or software that are central to the research but not yet described in published literature, software must be made available to editors and reviewers. We strongly encourage code deposition in a community repository (e.g. GitHub). See the Nature Portfolio guidelines for submitting code & software for further information.

Data

Policy information about availability of data

All manuscripts must include a data availability statement. This statement should provide the following information, where applicable:
- Accession codes, unique identifiers, or web links for publicly available datasets
- A description of any restrictions on data availability
- For clinical datasets or third party data, please ensure that the statement adheres to our policy

Code for Delphi and accompanying scripts and Jupyter notebooks are available on github.com/gerstung-lab/delphi. The model's checkpoint is available per UK Biobank's controlled access procedures with upload ID 7318.

UK Biobank data are available under restricted access through a procedure described at http://www.ukbiobank.ac.uk/using-the-resource/.

Danish registry data are available for use in secure, dedicated environments via application to the Danish Patient Safety Authority and the Danish Health Data Authority via https://sundhedsdatastyrelsen.dk/da/english/health_data_and_registers/research_services/apply.

Research involving human participants, their data, or biological material

Policy information about studies with human participants or human data. See also policy information about sex, gender (identity/presentation), and sexual orientation and race, ethnicity and racism.

Reporting on sex and gender	We use self-reported sex of participants as recorded in UKB field 31 (indicators for female and male) as an input to the model while training and to evaluate the performance of the model in the sex-stratified manner.
Reporting on race, ethnicity, or other socially relevant groupings	To assess model performance in subgroups, but not as a part of the data for model training, the following self-reported information was used:<br>- Ethnic background as available in field 21000 (UKB), participants grouped into 5 level groups (White, Mixed, Asian or Asian British, Black or Black British and Chinese)<br>- Index of multiple deprivation as available in field 26410 (combines information across seven domains including Income, Employment Derivation, Health and Disability, Education Skills and Training, Barriers to Housing and Services, Living Environment, and Crime)
Population characteristics	The distribution of the disease tokens over sex and age is shown in Supplementary Figure 12.
Recruitment	The was no recruitment for this study, the data was obtained from the UK Biobank and Danish National Patient Registry. We restrict Danish cohort to individuals aged 50-80 on 1st of January 2016, to obtain a similar age range as in the UK Biobank.
Ethics oversight	The UK Biobank has received approval from the National Information Governance Board for Health and Social Care and the National Health Service North West Centre for Research Ethics Committee 532 (Ref: 11/NW/0382). All UK Biobank participants gave written informed consent and were free to withdraw at any time. This research was conducted using the UK Biobank Resource under project 49978. All investigations were conducted in accordance with the tenets of the Declaration of Helsinki.<br><br>The use of the Danish National Patient Registry for validation of the UK Biobank results was conducted in compliance with the General Data Protection Regulation of the European Union and the Danish Data Protection Act. The analyses were conducted under the data confidentiality and information security policies of the Danish National Statistical Institute Statistics Denmark. The Danish Act on Ethics Review of Health Research Projects and Health Data Research Projects ("the Committee Act") do not apply to the type of secondary analysis of administrative data reported in this paper.

Note that full information on the approval of the study protocol must also be provided in the manuscript.

Field-specific reporting

Please select the one below that is the best fit for your research. If you are not sure, read the appropriate sections before making your selection.

☒ Life sciences ☐ Behavioural & social sciences ☐ Ecological, evolutionary & environmental sciences

For a reference copy of the document with all sections, see nature.com/documents/nr-reporting-summary-flat.pdf

Life sciences study design

All studies must disclose on these points even when the disclosure is negative.

Sample size	We used "train-validation-test" split that is standard for machine learning papers.<br><br>The models were trained on UK Biobank data for 402,786 (random 80%) individuals using data from birth until 30th of June 2020. For horizontal validation, data contains the remaining 100,636 (20%) individuals for the same period. Testing (also referred as longitudinal validation) was carried out using data for all individuals still alive by the cutoff date (471,057) and evaluated on disease incidence from 1st of July 2021 to 1st of July 2022.<br><br>To evaluate the model generalisation, we additionally tested it using Danish national patient registry, containing 1,931,630 individuals.
Data exclusions	For evaluating the model performance (AUC-ROC, SHAP values), only diseases with more than 25 entries in the UK Biobank validation dataset were used.
Replication	We performed 4 technical replicates of model training using different training/validation data splits, with all models performing similarly in terms of disease prediction AUC (Supplementary Figure 1)<br>Additionally, multiple models (n=486) were trained for hyperparameter search, the parameters varied included embedding dimension, context size, number or attention heads, number of transformer layers. Many models showed comparable performance in terms of validation cross-entropy loss, though some of them naturally were better, as finding those was purpose of this experiment.
Randomization	We randomly divided UK Biobank dataset to training and validation cohorts, using ratio 4:1. Randomisatition was not required to the Danish data, as it was used as a validation dataset, without splitting it to subgroups.
Blinding	Anonymised patient identifiers provided by the UK Biobank were used. We didn't perform any additional blinding for Danish data, as the analysis in paper is not a clinical trial.

Reporting for specific materials, systems and methods

We require information from authors about some types of materials, experimental systems and methods used in many studies. Here, indicate whether each material, system or method listed is relevant to your study. If you are not sure if a list item applies to your research, read the appropriate section before selecting a response.

Materials & experimental systems

n/a	Involved in the study	
☒	☐	Antibodies
☒	☐	Eukaryotic cell lines
☒	☐	Palaeontology and archaeology
☒	☐	Animals and other organisms
☒	☐	Clinical data
☒	☐	Dual use research of concern
☒	☐	Plants

Methods

n/a	Involved in the study	
☒	☐	ChIP-seq
☒	☐	Flow cytometry
☒	☐	MRI-based neuroimaging

Plants

Seed stocks	*Report on the source of all seed stocks or other plant material used. If applicable, state the seed stock centre and catalogue number. If plant specimens were collected from the field, describe the collection location, date and sampling procedures.*
Novel plant genotypes	*Describe the methods by which all novel plant genotypes were produced. This includes those generated by transgenic approaches, gene editing, chemical/radiation-based mutagenesis and hybridization. For transgenic lines, describe the transformation method, the number of independent lines analyzed and the generation upon which experiments were performed. For gene-edited lines, describe the editor used, the endogenous sequence targeted for editing, the targeting guide RNA sequence (if applicable) and how the editor was applied.*
Authentication	*Describe any authentication procedures for each seed stock used or novel genotype generated. Describe any experiments used to assess the effect of a mutation and, where applicable, how potential secondary effects (e.g. second site T-DNA insertions, mosiacism, off-target gene editing) were examined.*
